# Topping-Off Technology versus Posterior Lumbar Interbody Fusion in the Treatment of Lumbar Disc Herniation: A Meta-Analysis

**DOI:** 10.1155/2020/2953128

**Published:** 2020-01-13

**Authors:** Wei Wang, Xiangyao Sun, Tongtong Zhang, Siyuan Sun, Chao Kong, Shibao Lu

**Affiliations:** ^1^Department of Orthopaedics, Xuanwu Hospital Capital Medical University, Beijing 100053, China; ^2^National Clinical Research Center for Geriatric Diseases, Beijing 100053, China; ^3^Department of Orthopaedics, ChuiYang Liu Hospital, Tsinghua University, Beijing 100020, China; ^4^Department of Interdisciplinary, Life Science, Purdue University, West Lafayette, IN 47907, USA

## Abstract

The treatment effects of topping-off technique were still controversial. This study compared all available data on postoperative clinical and radiographic outcomes of topping-off technique and posterior lumbar interbody fusion (PLIF). PubMed, EMBASE, and Cochrane were systematically reviewed. Variations included radiographical adjacent segment disease (RASD), clinical adjacent segment disease (CASD), global lumbar lordosis (GLL), visual analogue scale (VAS) of back (VAS-B) and leg (VAS-L), Oswestry disability index (ODI), Japanese Orthopaedic Association (JOA) score, duration of surgery, estimated blood loss (EBL), reoperation rates, and complication rates. Sixteen studies, including 1372 cases, were selected for the analysis. Rates of proximal RASD (*P*=0.0004), distal RASD (*P*=0.03), postoperative VAS-B (*P*=0.0001), postoperative VAS-L (*P*=0.02), EBL (*P*=0.007), and duration of surgery (*P*=0.02) were significantly lower in topping-off group than those in PLIF group. Postoperative ODI after 3 years (*P*=0.04) in the topping-off group was significantly less than that in the PLIF group. There was no significant difference in the rates of CASD (*P*=0.06), postoperative GLL (*P*=0.14), postoperative ODI within 3 years (*P*=0.24), and postoperative JOA (*P*=0.70) and in reoperation rates (*P*=0.32) and complication rates (*P*=0.27) between topping-off group and PLIF. The results confirmed that topping-off technique could effectively prevent ASDs after lumbar internal fixation. However, this effect is effective in preventing RASD. Topping-off technique is more effective in improving the subjective feelings of patients rather than objective motor functions compared with PLIF. With the development of surgical techniques, both topping-off technique and PLIF are safe.

## 1. Introduction

Chronic low back pain (CLBP) is one of the main reasons for seeking medical treatment [1, 2]. Since Albee [3] and Hibbs [4] first reported intervertebral fusion, it had been considered the standard surgical treatment for patients suffering from CLBP caused due to lumbar degenerative disease, including lumbar disc herniation, lumbar spinal stenosis, and lumbar spondylolisthesis [5]. Although favorable clinical outcomes could be achieved, there were still a series of complications [6], the major of which is the acceleration of adjacent segment diseases (ASDs) due to the increase in range of motion (ROM) of adjacent segments [7]. In order to decrease ASDs after fusion, the concept of “dynamic stability” has been introduced; many different materials and technologies for dynamic stability have been put forward [8]. Khoueir et al. [8] had reported three types of posterior dynamic stabilization systems: interspinous process device (IPD), pedicle-based stabilization devices (PDS), and total facet replacement system. “Topping-off” technique is a hybrid dynamic stabilization system, which combined rigid fusion with dynamic nonfusion of adjacent segments such as IPD or PDS for the purpose of reducing hypermobility and overstress of the disk in the adjacent segment [9–13].

At the present study, there have been various flexible systems in spinal motion preservation technology. The topping-off technique is a newly developed surgical technique, which combines rigid fusion with an interspinous process device in the adjacent segment to prevent ASD [14]. However, the comparison between topping-off technique and fusion therapy in postoperative outcomes is controversial due to the lack of clear clinical evidences [13, 15, 16]. Most previous studies focused on the hypermobility at the adjacent segments of topping-off technique. Buric et al. [17] investigated the effect of long rigid fixation on adjacent levels with and without a transitional posterior dynamic stabilization at the caudal end. Their results showed an increased motion in the distal adjacent levels in all loading modes. This could easily lead to degeneration of multiple segments. However, Chen et al. [18] stated that topping-off surgery was able to maintain disc height and foraminal height than fusion surgery; however, due to natural disc degeneration, the parameters of Coflex segment were significantly decreased to the initial state compared to fusion group from one year postoperatively to last follow-up. They found that all the changes of these parameters did not cause any change of the clinical scores. Therefore, we conducted a meta-analysis to compare all available data on postoperative clinical and radiographic outcomes of topping-off technique and posterior lumbar interbody fusion (PLIF).

## 2. Materials and Methods

### 2.1. Search Strategy

The present review was conducted in accordance with Preferred Reporting Items for Systematic Reviews and Meta-Analyses (PRISMA) Statement [19]. A comprehensive literature search was performed by an experienced librarian. Relevant studies were identified in PubMed, EMBASE, Cochrane databases, and CNKI from 1980 to October 2019. The following medical subject headings and keywords were used: “hybrid stabilization,” “topping off,” “hybrid stabilization device,” “dynamic hybrid,” “hybrid fixation,” “fusion,” “lumbar,” “lumbar degenerative disease,” “adjacent segment degeneration,” and “adjacent segment disease.” The computer searches were supplemented by manual searches of all retrieved research and review reference lists.

### 2.2. Inclusion Criteria and Exclusion Criteria

Studies that meet the following criteria included the following: patient cohorts underwent PLIF or topping-off surgery for lumbar degenerative disease frustrated conservative treatment; patients of the two groups have the same baseline of preoperative radiograph; clinical evaluations were followed up for no less than 12 months. Exclusion criteria were therapies for tumors, infections, and revision surgery or congenital malformations; similarly, biomechanical studies and nonhuman or in vitro studies were excluded; abstracts, case reports, expert opinions, and noncomparative study were also excluded.

### 2.3. Data Extraction

All data are extracted from the text, pictures, and tables of the articles. Basic characteristics included age, gender, duration, study design, and enrolled number. Primary outcomes included radiographical and clinical adjacent segment disease (RASD, CASD), global lumbar lordosis (GLL), visual analogue scale (VAS) of back and leg (VAS-B, VAS-L), Oswestry disability index (ODI), and Japanese Orthopaedic Association (JOA) score. The other outcomes included duration of surgery, estimated blood loss (EBL), reoperation rates, and complication rates. Two authors independently extracted data from the included studies. Other authors will be invited to participate in the discussion until a consensus opinion is reached if there is disagreement about the outcome.

### 2.4. Quality Assessment

Two review authors independently assessed the quality of the cohort studies by using the Newcastle–Ottawa Scale (NOS) [20]. A full score of 9 stars and a score of 7 or more are considered to be an excellent quality study.

### 2.5. Data Analysis

The statistical analysis of the results of the study was performed by RevMan5.3 software. In summary statistics, odds ratios (OR) and standardized mean difference (SMD) with 95% confidence intervals (CI) are used for dichotomous data and continuous data. Moreover, heterogeneity across trials was explored using the result of chi-squared test and *I*^2^ statistic. Random effect model was used if there is a significant heterogeneity assumed as *P* value less than 0.05 and *I*^2^ > 50%. Otherwise, data were pooled by using the fixed effect model. If there is a potential heterogeneity, we performed subgroup analysis and sensitivity tests in conjunction with possible clinical realities. *P* value less than 0.05 was considered of statistical significance.

## 3. Results

### 3.1. Study Characteristics

**Figure 1:**
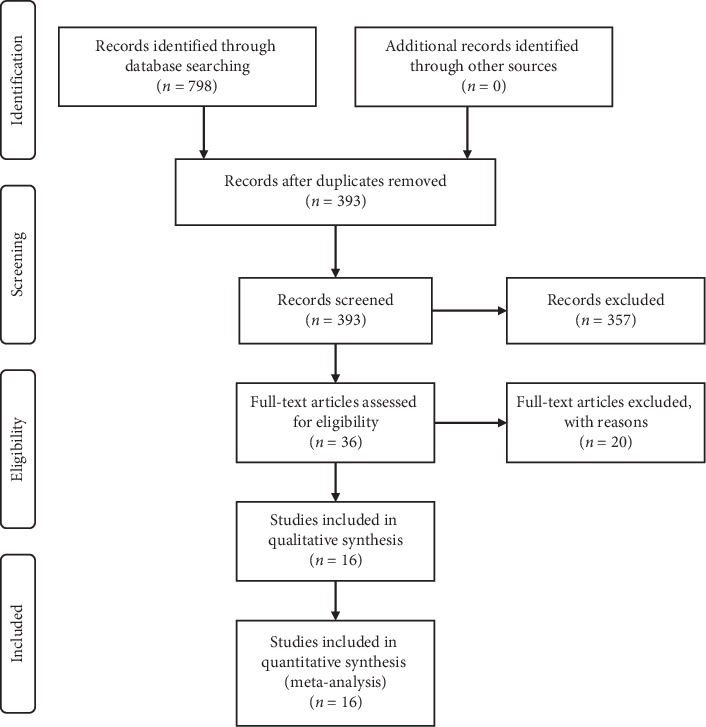
Flow chart showing identification and selection of cases.

A total of 798 references were identified in the initial examination. After exclusion of duplicate or irrelevant references, 393 articles were retrieved. Ultimately, 16 studies [11, 14, 16, 18, 21–32], including 1372 cases, were selected for analysis after two authors reviewed the full text according to the inclusion and exclusion criteria (Figure 1).  Table 1 shows the characteristics of the included studies.

### 3.2. Adjacent Segment Disease

**Figure 2:**
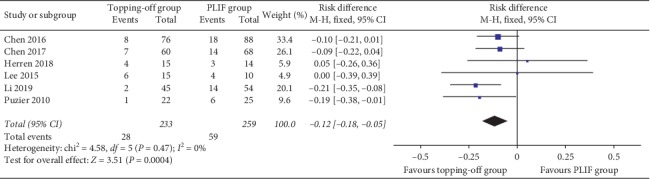
Forest plot of proximal RASD.

**Figure 3:**
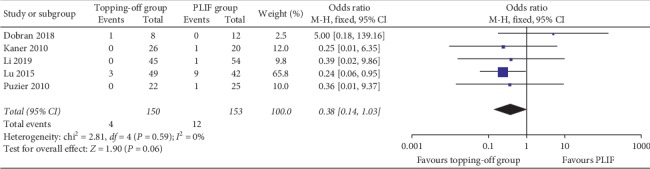
Forest plot of CASD.

Six studies [11, 16, 18, 21, 26, 28] reported the incidence of proximal RASD (Figure 2). The fixed effect model was applied considering that there is no significant heterogeneity between the two groups (*I*^2^ = 0%). The incidence of proximal RASD in topping-off group was significantly less than that in PLIF group (OR −0.12; 95% CI −0.18, −0.05; *I*^2^ = 0%; *P*=0.0004). The incidence of distal RASD was discussed in three studies [11, 18, 28]. Because there is no significant heterogeneity between the two groups (*I*^2^ = 0%), fixed effect model was applied in this analysis. No significant between-group difference was found in this analysis (OR 0.27; 95% CI 0.08, 0.89; *I*^2^ = 0%; *P*=0.03). Five articles [16, 23, 26, 32, 33] reported the incidence of CASD (Figure 3). The fixed effect model was applied considering that there is no significant heterogeneity between the two groups (*I*^2^ = 0%). No between-group difference was found (OR 0.38; 95% CI 0.14, 1.03; *I*^2^ = 0%; *P*=0.06).

### 3.3. Global Lumbar Lordosis

**Figure 4:**
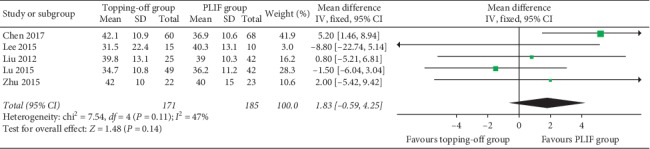
Forest plot of postoperative GLL.

Postoperative GLL was evaluated in 5 studies [11, 14, 23, 25, 28]. The fixed effect model was applied considering that there is no significant heterogeneity between the two groups (*I*^2^ = 0%). No significant between-group difference was found in fixed effect model (SMD −1.83; 95% CI −0.59, 4.25; *I*^2^ = 47%; *P*=0.14, Figure 4).

### 3.4. Clinical Scoring System

**Figure 5:**
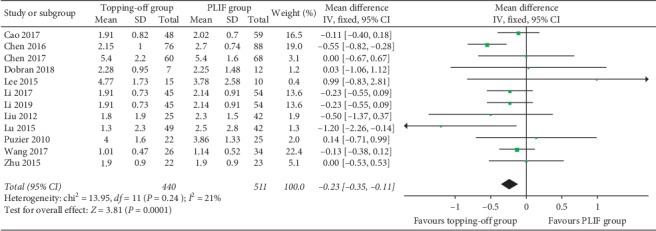
Forest plot of postoperative VAS-B.

**Figure 6:**
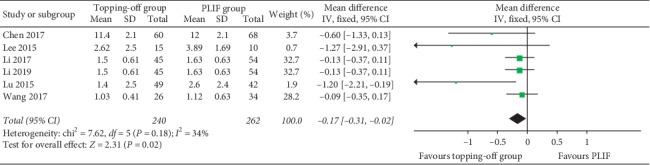
Forest plot of postoperative VAS-L.

VAS-B was documented in 12 articles [11, 14, 16, 18, 23, 25–30, 32] (Figure 5). The fixed effect model was applied considering that there is no significant heterogeneity between the two groups (*I*^2^ = 21%). VAS-B in the topping-off group was significantly less than that in the PLIF group (SMD −0.23; 95% CI −0.35, −0.11; *I*^2^ = 21%; *P*=0.0001). Six studies [11, 23, 26–28, 30] discussed VAS-L (Figure 6). Fixed effect model was used in this analysis (*I*^2^ = 34%). VAS-L in the topping-off group was significantly less than that in the PLIF group (SMD −0.17; 95% CI −0.31, −0.02; *I*^2^ = 34%; *P*=0.02).

**Figure 7:**
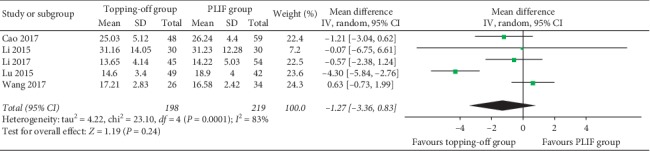
Forest plot of postoperative ODI within 3 years.

**Figure 8:**
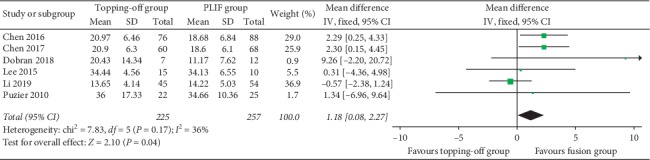
Forest plot of postoperative ODI after 3 years.

Five studies [22, 27, 29–31] evaluated postoperative ODI within 3 years (Figure 7). Because there was a significant heterogeneity in this analysis (*I*^2^ = 83%), random effect model was used in this analysis. No significant between-group difference was found (SMD −1.27; 95% CI −3.36, 0.83; *I*^2^ = 83%; *P*=0.24). Six studies [23, 27, 29–31] reported postoperative ODI after 3 years. No significant heterogeneity (*I*^2^ = 36%) was found, and the fixed effect model was used in this analysis. Result showed that postoperative ODI was significantly less in PLIF group than that in the topping-off group (SMD 1.18; 95% CI 0.08, 2.27; *I*^2^ = 36%; *P*=0.04) (Figure 8). Four studies [14, 25, 28, 30] discussed postoperative JOA. No between-group difference could be found in this analysis (SMD −0.34; 95% CI −2.09, 1.40; *I*^2^ = 76%; *P*=0.70).

### 3.5. Intraoperative Parameters

**Figure 9:**
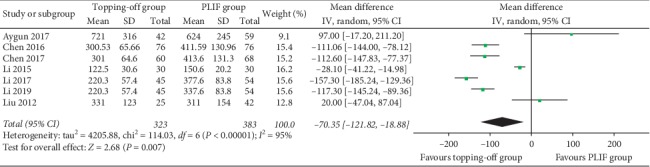
Forest plot of EBL.

**Figure 10:**
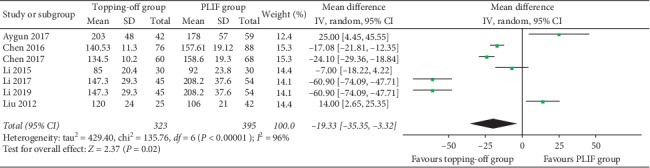
Forest plot of duration of surgery.

EBL was reported in 7 studies [18, 22, 25, 28, 30–32]. Random effect model was used in this analysis, because a significant heterogeneity could be found (*I*^2^ = 95%). No between-group significance could be found in EBL (SMD −70.35; 95% CI −121.82, −18.88; *I*^2^ = 95%; *P*=0.007) (Figure 9). Duration of surgery was documented in 7 studies [18, 22, 25, 26, 28, 30, 31]. Random effect model was used in this analysis, considering that there was a significant heterogeneity between these studies (*I*^2^ = 96%). Duration of surgery in the topping-off group was significantly less than that in the PLIF group (SMD −19.33; 95% CI −35.35, −3.32; *I*^2^ = 34%; *P*=0.02) (Figure 10).

### 3.6. Complications

**Figure 11:**
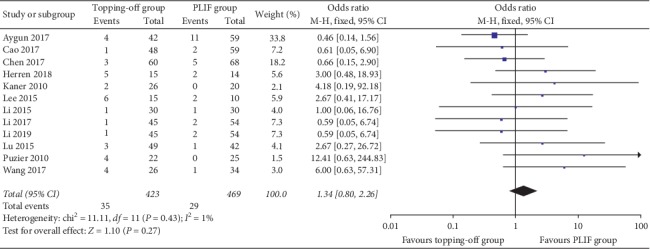
Forest plot of complication rates.

Twelve studies [11, 16, 21–23, 26–30, 33] including 293 patients reported incidences of complications. Fixed effect model was used in this analysis without a significant heterogeneity (*I*^2^ = 1%). No significant between-group difference could be found in the results (OR 1.34; 95% CI 0.80, 2.26; *I*^2^ = 1%; *P*=0.27) (Figure 11). Dural tear rates were reported in five studies [22, 23, 28, 29, 31]. Fixed effect model was used in this evaluation (*I*^2^ = 0%), and no significant difference was found between topping-off group and PLIF group (OR 0.89; 95% CI 0.29, 2.71; *I*^2^ = 0%; *P*=0.84). Five studies discussed the infection rates [22, 23, 26, 28, 30]. No significant between-group difference could be found in the results (OR 0.52; 95% CI 0.14, 1.89; *I*^2^ = 0%; *P*=0.32). Three articles [11, 16, 22] discussed the pseudoarthrosis rates. Fixed effect model was used in this analysis (*I*^2^ = 0%). No between-group significance could be found in the results (OR 1.09; 95% CI 0.36, 3.27; *I*^2^ = 0%; *P*=0.88). Incidences of screw loosening were discussed in 3 articles [16, 20, 21]. Fixed effect model was used in this evaluation (*I*^2^ = 0%), and no significant difference was found between topping-off group and PLIF group (OR 1.86; 95% CI 0.46, 7.57; *I*^2^ = 0%; *P*=0.39). Two articles [16, 21] evaluated implant breakage rates. There was no significant difference between topping-off group and PLIF group (OR 2.15; 95% CI 0.39, 11.81; *I*^2^ = 57%; *P*=0.38). Hematoma formation was evaluated in two articles [26, 30]. No significant between-group difference was found in this analysis (OR 1.20; 95% CI 0.17, 8.73; *I*^2^ = 0%; *P*=0.85).

**Figure 12:**
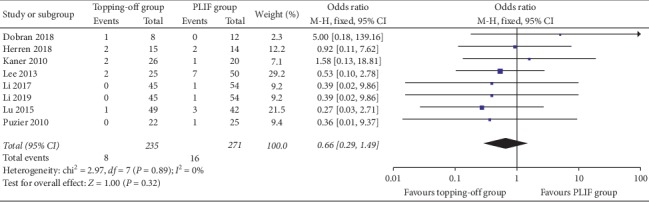
Forest plot of reoperation rates.

Eight studies discussed reoperation rates [16, 20, 22, 24, 26, 30, 32, 33]. There was no significant heterogeneity in this analysis (*I*^2^ = 0%). Fixed effect model was used in this evaluation. No significant between-group difference was found in the results (OR 0.50; 95% CI 0.17, 1.43; *I*^2^ = 0%; *P*=0.19) (Figure 12).

## 4. Discussion

The “topping-off” technique uses dynamic fixation to prevent the proximal adjacent segments from degenerating, which can reduce the incidence of ASD [34]. At the same time, the elastic fixation segment can act as a partially active buffer between fused segments and proximal mobile segments. Whether ASD is a natural evolution of an aging spine or a consequence of spinal fusion is still controversial [35, 36]. Nevertheless, many risk factors for ASD, such as age over 50 years [37, 38], sagittal imbalance [39], increased length of fusion, and surgical approaches, have been identified in previous studies [40].

Considering that there is still a lack of uniformity in the criteria of RASD, we summarized the similarities in previous studies as follows: dynamic angulation of the interspinous space less than 10°, loss of intervertebral disc height, and spondylolisthesis increase more than 3 mm [11, 14, 18, 22–24, 41]. The results of our research showed that the incidence of proximal RASD and distal RASD in the PLIF group was significantly higher than that in the topping-off group. These results were in consistent with previous studies [13, 42]. However, no significant between-group difference was found in CASD, which indicated that topping-off technique was more effective in preventing radiographical parameters. This may be explained by the decrease of compensatory mechanisms in the segmental range of motion in the topping-off group [34, 43]. Our results showed that postoperative GLL was similar in the topping-off group and PLIF group. This indicates that even though there is an expansion effect in the topping-off technology, this effect can be negligible in global spine compared with PLIF; correction of GLL may not be an advantage of topping-off technology [21, 34].

The scores of JOA, ODI, and VAS are important indexes to judge the recovery of lumbar functions and the curative effects after operation. It has been reported that topping-off surgery can achieve a good clinical improvement even in the long-term follow-up [2, 8, 44]. In this analysis, VAS-B and VAS-L in the topping-off group were significantly less than those in the PLIF group; in addition, postoperative ODI in the topping-off group was significantly higher than that in the PLIF group after 3 years; however, postoperative ODI within 3 years and JOA score were similar in both groups. This may be associated with the increasing age and the complications of dynamic equipment [45]. JOA score focused more on the objective motor functions of patients while VAS and ODI give more weight to subjective feelings of patients [14, 25]. This indicates that topping-off technique is more effective in improving subjective feelings of patients rather than objective motor functions compared with PLIF. This difference will be more obvious 3 years after the operation.

Our study showed that EBL and duration of surgery in topping-off group were significantly less than those in PLIF group. This is because insertion of dynamic implants does not need the additional exposure of anatomical structures, which can save the operation time and decrease the EBL [8]. These results need to be interpreted carefully because there are many confounding factors.

The potential of topping-off technique in decreasing the incidence of complications after fusion surgery remains controversial. The common complications after topping-off surgery are screw loosening, screw fracture, and spinous process avulsion fracture [16, 21, 23, 24]. It has been reported that the most common complications are screw loosening for HSDs and spinous process fracture for IPSs [46, 47]. However, our study showed that no significant difference between topping-off group and PLIF group was found in the rates of complications, such as dural tear, infection, implant loosening, pseudoarthrosis, and implant breakage. The discovery of “halo zone” in dynamic stabilization systems based on plain radiographs for the diagnosis of implant-related complications has been reported previously [45]. According to this theory, the forces conveyed from the dynamic implant can increase the stress on rigid fixation over time, and then implant-associated adverse events will occur. In contrast, our results showed that this effect did not affect the application of the topping-off technique compared with PLIF. Hydroxyapatite coated pedicle screws can be introduced to prevent implant-related complications [48]. It has been reported that hydroxyapatite can promote bone deposition on the implant surface and promote the formation of direct chemical bonds between the implant and the bone interface, which may reduce the complication rates in patients [35, 49].

Several limitations or drawbacks could be ignored in this meta-analysis. First, only one article in the included studies is a randomized controlled trial. As a result, there are inherent limitations associated with the risks of reporting or selection bias. Second, lumbar degeneration is a series of diseases in which the overall outcome could vary depending on specific diagnosis, such as intervertebral disc herniation, stenosis, and spondylolisthesis. Third, different types of dynamic devices used in adjacent segments may affect the outcomes. Fourth, recent treatments of degenerative disc diseases and instabilities have used various approaches, such as anterior lumbar interbody fusion (ALIF), PLIF, and transforaminal lumbar interbody fusion (TLIF). However, according to the inclusion and exclusion conditions, no relevant articles discussing ALIF or TLIF can be included in our analysis. This will narrow the scope of our conclusions. Therefore, more randomized controlled trials are still needed to draw a more convincing conclusion.

## 5. Conclusions

Our study confirms previous findings that topping-off could effectively delay the ASD process after lumbar internal fixation. However, this effect is effective in preventing RASD. Topping-off technique is more effective in improving subjective feelings of patients rather than objective motor functions compared with PLIF. This difference will be more obvious 3 years after the operation. With the development of surgical techniques, both topping-off technique and PLIF are safe.

## Figures and Tables

**Table 1:** Characteristics of included studies.

Study	Year	Country	Study type	Quality (NOS)	LoE	Device	Patients (F/M)		Age (years)		Follow-up (months)	Segments
							PLIF	Topping-off	PLIF	Topping-off		
Putzier et al. [16]	2010	Germany	Pro	7	II	Dynesys	30 (16/14）	30 (13/17)	44.6	44.9	76.4	1
Kaner et al. [33]	2010	Turkey	Pro	6	II	Coflex	20	26	—	—	40	—
Liu et al. [25]	2012	China	Retro	7	III	Coflex	48 (20/28)	31 (11/20)	41.5	44.6	24	1
Lee et al. [24]	2013	Korea	Retro	7	III	DIAM	50 (20/30)	25 (10/25)	65.9 ± 8.5	65.4 ± 8.7	46.8	1
Lee et al. [11]	2015	Korea	Retro	8	III	DTO/Nflex	10 (5/5)	15 (11/4)	63.9 ± 7.8	60.7 ± 8.3	48	2
Zhu et al. [14]	2015	China	Retro	6	III	Wallis	23 (12/11)	22 (8/14)	40	44.5	24	1
Lu et al. [23]	2015	China (Taiwan)	Retro	7	III	DIAM	42 (14/28)	49 (16/33)	64.5 ± 7.2	59.1 ± 8.6	41.5	2–4
Li and Wu [31]	2015	China	Pro	7	III	Coflex	30 (11/19)	30 (12/18)	46.4	47.3	20 (12–42)	1
Chen et al. [18]	2016	China	Retro	7	III	Coflex	88 (34/54)	76 (28/48)	58.31 ± 4.6	57.34 ± 5.1	47.2	1
Aygun et al. [22]	2017	Turkey	Retro	6	III	Cosmic	59 (34/25)	42 (19/23)	54.2 ± 5.11	52 ± 6.02	79	1–5
Wang et al. [27]	2017	China	Retro	7	III	K-rod	34 (15/19)	26 (12/14)	44.9	46.7	17.1	2
Chen et al. [28]	2017	China	Retro	8	III	Coflex	68 (26/42)	60 (22/38)	56.2 ± 4.2	54.2 ± 4.1	50	2
Cao et al. [29]	2017	China	Retro	6	III	Coflex	59 (25/34)	48 (22/26)	39.3	38	30	1
Li et al. [30]	2017	China	Retro	6	III	Coflex	54 (29/25)	45 (22/21)	63.7	61.5	35.2	1–2
Dobran et al. [32]	2018	Italy	Retro	5	III	NFlex	12	9	63	66	78	1–2
Herren et al. [21]	2018	Germany	RCT	9	I	Dynesys	14 (8/6)	15 (6/9)	61.78 (34–76)	60.9 (47–80)	37.68 (1.38–72)	1–5
Li et al. [26]	2019	China	Retro	6	III	Coflex	54 (29/25)	45 (24/21)	53.5 (46–59)	65.7 (60–75)	56.4 ± 105.6 (36–37)	2

Notice: NOS = Newcastle–Ottawa Scale score; RCT = randomized controlled trial; Retro = retrospective cohort study; Pro = prospective cohort study; PLIF = posterior lumbar interbody fusion.

## Abbreviations

CLBP: Chronic low back pain
ASDs: Adjacent segment diseases
ROM: Range of motion
IPD: Interspinous process device
PDS: Pedicle-based stabilization devices
PLIF: Posterior lumbar interbody fusion
PRISMA: Preferred Reporting Items for Systematic Reviews and Meta-Analyses
RASD: Radiographical adjacent segment disease
CASD: Clinical adjacent segment disease
GLL: Global lumbar lordosis
VAS: Visual analogue scale
VAS-B: VAS of back
VAS-L: VAS of leg
ODI: Oswestry disability index
JOA: Japanese Orthopaedic Association
EBL: Estimated blood loss
NOS: Newcastle–Ottawa Scale
OR: Odds ratios
SMD: Standardized mean difference
CI: Confidence intervals
ALIF: Anterior lumbar interbody fusion
TLIF: Transforaminal lumbar interbody fusion.
